# Prevalence of *Hemoplasma* spp. positivity in potential feline blood donors and study of the association with selected clinical variables

**DOI:** 10.1111/jvim.17119

**Published:** 2024-05-27

**Authors:** Elodie Roels, Chiara Debie, Sophie Giraud, Rui Ferreira, Kris Gommeren

**Affiliations:** ^1^ Department of Clinical Sciences, FARAH, Faculty of Veterinary Medicine University of Liège Liège Belgium; ^2^ Small Animal Department, Faculty of Veterinary Medicine Ghent University Merelbeke Belgium; ^3^ Banco Sangue Animal (BSA)—Animal Blood Bank Porto Portugal

**Keywords:** blood bank, blood donation, blood transfusion, cats, *Mycoplasma haemofelis*

## Abstract

**Background:**

Hemotropic mycoplasmas, hemoplasmas, are epi‐erythrocytic parasitic bacteria that can be transmitted through blood transfusion.

**Objectives:**

To study the prevalence of hemoplasma infection of potential feline blood donors and investigate the association between *Hemoplasma* spp. quantitative polymerase chain reaction (qPCR) positivity in blood units and selected variables.

**Animals:**

Seven thousand five hundred seventy‐three blood units from 4121 privately‐owned potential donor cats.

**Methods:**

Retrospective observational cross‐sectional study. The Banco Sangue Animal (BSA)—Animal Blood Bank medical database was reviewed for all feline donations performed in 2022 in Portugal, Spain, and Belgium. Baseline characteristics and results of blood‐borne pathogens screening tests were extracted from the medical records.

**Results:**

Two hundred twelve of 4034 Portuguese donor cats and 2 of 70 Spanish donor cats tested positive for *Hemoplasma* spp. qPCR in 2022 leading to an overall estimated prevalence of 5.2% (95% CI: 4.5%‐5.9%) in potential blood donors. Using multivariable generalized estimation equation models, *Hemoplasma* spp. qPCR was more often positive among blood units issued from male cats (OR = 1.9, 95% CI: 1.4‐2.6, *P* < .0001), units positive for FeLV (OR = 2.8, 95% CI: 1.4‐5.6, *P* = .0023), and units collected in winter months (OR = 2.5, 95% CI: 1.7‐3.6, *P* < .0001).

**Conclusions and Clinical Importance:**

This study underscores the importance of *Hemoplasma* spp. and other relevant blood‐borne pathogens screening at every donation. Implementing stringent screening protocols is crucial to mitigate the risk of hemoplasma transmission via blood transfusions, thereby safeguarding the health and welfare of cats receiving transfusions.

**Abbreviations**
95% CI: 95% confidence interval
BSA: Banco Sangue Animal
CMhm: *Candidatus* Mycoplasma haemominutum
CMt: *Candidatus* Mycoplasma turicensis
FeLV: feline leukemia virus
FIV: feline immunodeficiency virus
GEE: generalized estimation equation
Mhf: *Mycoplasma haemofelis*
PCR: polymerase chain reaction
qPCR: quantitative polymerase chain reaction
RH: relative humidity

## 1 INTRODUCTION

Blood products are transfused in feline medicine for the management of anemias, coagulopathies and other conditions.^1^ Hemotropic mycoplasmas, also called hemoplasmas, are epi‐erythrocytic, wall‐less, gram‐negative bacteria that can be transmitted via red blood cell transfusions with potentially clinically important consequences.^2^, ^3^ Accordingly, current guidelines advise to screen all donor cats for *Hemoplasma* spp. by conventional or quantitative polymerase chain reaction (qPCR).^4^, ^5^, ^6^ The 3 most common feline hemoplasmas are *Mycoplasma haemofelis* (Mhf), *Candidatus* Mycoplasma haemominutum (CMhm) and *Candidatus* Mycoplasma turicensis (CMt).^6^, ^7^ Feline blood units positive for Mhf should not be transfused and infected donors should be removed from the donor program, while a clear consensus on CMhm and CMt has not been provided because of the lack of evidence for relevant clinical disease after transmission.^4^, ^5^, ^6^ The prevalence of hemoplasmas in the general population of cats vary from 10.6% to 43.4%, depending on the geographical area and the type of study sample.^2^, ^8^, ^9^, ^10^, ^11^, ^12^, ^13^, ^14^, ^15^, ^16^ Male sex, adult age, nonpedigree breed, outdoor access, blood genotype, and coinfection with retroviruses (feline immunodeficiency virus [FIV] and/or feline leukemia virus [FeLV]) are associated with *Hemoplasma* spp. PCR positivity.^2^, ^8^, ^9^, ^10^, ^11^, ^12^, ^13^, ^14^, ^15^, ^16^, ^17^, ^18^, ^19^, ^20^ *Hemoplasma* spp. prevalence is higher during the warmer months of the year in some studies.^9^, ^16^

To date, 2 studies have investigated the prevalence of hemoplasmas in feline blood donors.^21^, ^22^ In a study performed in 2006 in the United States, 15/118 (12.7%) of community‐sourced blood donor cats were positive for either Mhf and/or CMhm PCRs.^22^ In a second study published in 2021, 181/4880 (3.7%) cats eligible to become blood donors (healthy cats tested negative for retroviral infection on rapid screening test) in Spain and Portugal were positive for *Hemoplasma* spp. conventional PCRs.^21^

As information on the prevalence of hemoplasmas in feline blood donors is sparse, the primary aim of this study was to assess the prevalence of hemoplasmas in privately‐owned potential feline blood donors from Portugal, Spain and Belgium that donated blood at least once in 2022. To improve the level of confidence on previously published data regarding factors associated with hemoplasmas infection, a second aim was to investigate the association between *Hemoplasma* spp. qPCR positivity in feline blood donor units and the following selected variables: age, sex, breed, blood phenotype, geographical area, retroviral positivity, and season of blood donation.

## 2 MATERIALS AND METHODS

### 2.1 Animals and study design

The study design was a retrospective, cross‐sectional, observational study. Medical information on all feline blood donations performed during 2022 was retrospectively collected from the Banco Sangue Animal (BSA)—Animal Blood Bank medical records database searching over a 12‐month period (from 1st January 2022 to 31st December 2022). This study was conducted according to the European legislation on the protection of animals used for scientific purposes (2010/63/EU).

The study sample consisted of healthy cats that donated blood at least once in 2022 in a BSA—Animal Blood Bank center (https://bsanimal.pt). Cats were privately‐owned indoor with the possibility of having restricted supervised outdoor access. Cats were up to date with deworming and vaccination. Cats were either housed alone or with congeners. Stray cats, shelter cats or colony cats were not included in the blood donation program. Cats positive for FIV and/or FeLV on rapid screening test (Witness FeLV‐FIV, Zoetis, EU/International) or having a hemoglobin concentration below 10 g/dL (reference range: 10‐18 g/dL) at their initial visit when entering the blood donation program were excluded before donation and were not included in the present study. Full details on inclusion criteria for feline blood donors are reported elsewhere.^21^ Informed owner consent was obtained before blood donation. All feline blood donors had a comprehensive history taken and a complete physical examination performed by a veterinarian to ensure good health status before each donation. A complete hematological and biochemical blood analysis was done at inclusion in the program and then once a year at the first donation of the year to ensure adequate health status.

### 2.2 Blood‐borne pathogens screening

The screening tests results for blood‐borne pathogens were retrospectively evaluated from the laboratory records. All data extracted were obtained from routine procedures performed at the BSA—Animal Blood Bank.

After each blood donation, a separate blood sample was collected for blood‐borne pathogens screening using the different tests that are detailed below; all tests were run concurrently. Blood samples from each unit were tested for FeLV antigens and FIV antibodies using serological tests (VetLine FIV ELISA and FeLV antigen, NovaTec Immunodiagnostic GmbH, Germany), for FeLV provirus using qPCR (EXOone Feline Leukemia Virus oneMIX qPCR kit, Exopol, Spain), and for *Hemoplasma* spp. using qPCR (EXOone Haemoplasma spp. oneMIX qPCR kit, Exopol, Spain). Total DNA was extracted from blood samples using a commercially available kit (MagNA Pure Compact Nucleic Acid Isolation Kit I, Roche Diagnostics GmbH, Germany). *Hemoplasma* spp. qPCR was performed using a commercially available kit (EXOone Haemoplasma spp. oneMIX qPCR kit, Exopol, Spain) in accordance with the manufacturer's instructions (EXOone Haemoplasma spp. oneMIX qPCR kit, Exopol, Spain). The laboratory standard of physical separation of DNA extraction steps and DNA amplification steps was applied. An endogenous control (housekeeping gene) was used for evaluation of adequate sampling, nucleic acid extraction and PCR run (absence of PCR inhibitors), and a specific synthetic *Hemoplasma* spp. target DNA sequence was used for positive control and relative quantification; both controls were provided by the manufacturer (EXOone Haemoplasma spp. oneMIX qPCR kit, Exopol, Spain). Molecular grade water was used as a negative control. Endogenous, positive, and negatives controls were tested along with the samples at each PCR run. The total reaction volume was 20 μL containing 5 μL of template DNA and 15 μL of HAEM oneMIX (EXOone Haemoplasma spp. oneMIX qPCR kit, Exopol, Spain). Thermal cycler conditions of the PCR thermocycler (Real time LightCycler 480, Roche, Switzerland) system consisted of 5 minutes at 95°C and 42 cycles of 95°C for 15 seconds and 60°C for 60 seconds (EXOone Haemoplasma spp. oneMIX qPCR kit, Exopol, Spain). Blood units positive for at least 1 of the pathogens tested were immediately discarded and not used for blood donations. FIV, FeLV and/or *Hemoplasma* spp. positive cats were permanently removed from the blood donation program. A follow‐up blood‐borne pathogens screening after removal from the program was possible on owner request; but cats were not allowed to donate blood nor reenter the program even if a negative result was obtained at recheck.

### 2.3 Statistical analysis

Statistical analyses were performed using commercially available software (SAS version 9.4 for Windows, SAS Institute, Inc, Cary, North Carolina). Continuous variables were reported as median and interquartile range, and categorical data as number and percentage. For prevalence estimation, a cat was considered positive for *Hemoplasma* spp. qPCR if at least 1 of its blood units tested positive during the study period (year 2022). Prevalence was estimated using extract 95% confidence intervals (95% CI) for binomial proportions. An overall prevalence estimation was calculated from the entire study sample. Prevalence was also calculated per country and per Portuguese regions (Alentejo, Algavre, Centro, Lisboa, and Norte). Portuguese regions were identified by the cat's owner's postal addresses. Comparison of prevalence between Portuguese regions was conducted with a chi‐squared test. Univariable and multivariable generalized estimation equation (GEE) models were used to analyze the association between *Hemoplasma* spp. qPCR positivity in blood units and the following selected variables: age, gender, breed (pedigree or nonpedigree breed), blood phenotype, positivity for FeLV or FIV, Portuguese region, and seasonality (determined according to the blood donation date: Spring from 20th March until 20th June 2022, Summer from 21st June until 22nd September 2022, Autumn form 23rd September until 20th December 2022 and Winter from 01st January until 19th March 2022 and from 21st December until 31st December 2022). GEE models were used to account for repeated blood donations on a same cat. Univariable GEE analysis was used to estimate the crude odds ratios (ORs) and 95% CIs of clinical and epidemiological variables for *Hemoplasma* spp. qPCR positivity. The variables with a *P* < .1 in the univariable GEE model were selected into the multivariable GEE model. Crude and adjusted ORs (95% CIs) and *P*‐value were presented. Missing data were not replaced, and calculations were done on the maximum number of data available. Statistical significance was set at a *P* < .05.

## 3 RESULTS

### 3.1 Study sample

A total of 7573 blood donations from 4121 privately‐owned healthy potential feline donor cats, with a median age of a 3.9 years (2.4‐6.0) were retrospectively included. The baseline characteristics of potential blood donor cats are detailed in Table 1. There was an equal distribution of male and female gender among donor cats (Table 1). Information regarding the breed was missing for many included individuals (n = 2851, 69.2%). Among cats for which the breed was specified in the medical records (n = 1270, 30.8%), 821 (64.6%) were domestic shorthair (categorized as nonpedigree breed for the purpose of the study; Table 1); the remainder were American Curl (n = 1, 0.1%), Bengal (n = 7, 0.55%), Bobtail (n = 2, 0.16%), British short hair (n = 62, 4.9%), Maine Coon (n = 67, 5.3%), Munchkin (n = 2, 0.2%), Norwegian (n = 140, 11.0%), Persian (n = 67, 5.3%), Ragdoll (n = 13, 1.0%), Russian Blue (n = 1, 0.1%), Scottish fold (n = 14, 1.1%), Scottish straight (n = 30, 2.4%), Siamese (n = 26, 2.0%), Somali (n = 1, 0.1%), Sphynx (n = 13, 1.0%), and Toyger (n = 3, 0.2%). Most cats had blood phenotype A (n = 3976, 96.5%) and originated from Portugal (n = 4034, 97.9%; Table 1). Around half of the included cats (n = 2125, 51.6%) donated blood more than once in 2022 (Table 1). There was an equal distribution of blood donation across the seasons throughout the year with 1838 (24.3%) donations performed in Winter, 1822 (24.1%) in Spring, 1963 (25.9%) in Summer and 1950 (25.7%) in Autumn.

**TABLE 1:** Baseline characteristics of potential feline blood donors that donated blood in 2022 (n = 4121).

Variables	Total number (nonmissing data)	Subcategories	Number (%)
Sex	4121	Males	1928 (46.8)
		Females	2193 (53.2)
Breed	1270	Pedigree	449 (35.4)
		Nonpedigree	821 (64.6)
Blood type	4119	A	3976 (96.5)
		B	125 (3.0)
		AB	18 (0.4)
Country	4121	Belgium	17 (0.4)
		Spain	70 (1.7)
		Portugal	4034 (97.9)
Region (Portugal)	3959	Alentejo	24 (0.6)
		Algavre	3 (0.1)
		Centro	837 (21.1)
		Lisboa	968 (24.5)
		Norte	2127 (53.7)
Number of blood donations per cat	4121	1	1996 (48.4)
		2	1099 (26.7)
		3	725 (17.6)
		4	301 (7.3)

### 3.2 Prevalence estimation

Prevalence of hemoplasmas was estimated per country and per Portuguese region and was defined as the proportion of *Hemoplasma* spp. qPCR positive cats among the total number of cats that donated blood in 2022. As stated above in the statistical method section, a cat was considered positive for *Hemoplasma* spp. if a positive qPCR result was obtained during the studied period. Two hundred twelve Portuguese cats tested positive at least once for *Hemoplasma* spp. by qPCR leading to an estimated prevalence of 5.3% (95% CI: 4.6%‐5.9%) for *Hemoplasma* spp. in Portugal in 2022. Two Spanish cats had a positive *Hemoplasma* spp. qPCR once in 2022 leading to an estimated prevalence 2.9% (95% CI: 0.0%‐6.8%) for *Hemoplasma* spp. in Spain in 2022. No Belgian cats tested positive for *Hemoplasma* spp. qPCR; therefore, estimated prevalence was not applicable. An overall prevalence of 5.2% (95% CI: 4.5%‐5.9%) for *Hemoplasma* spp. was estimated using the pooled sample of all potential feline blood donors. Estimated prevalence by Portuguese regions did not significantly differ (*P* = .28; Table 2). Among Portuguese potential blood donor cats that were positive for *Hemoplasma* spp. via qPCR (n = 212) in 2022, 30 cats had their blood tested for blood‐borne pathogens >1 time: 26 cats were negative first then subsequently tested positive, 3 cats were positive on 2 occasions, and 1 cat was initially positive and subsequently tested negative. Repeated testing in *Hemoplasma* spp. positive cats was performed on the request of the owners; these cats were excluded from the blood donor pool and did not donate blood at the retesting occasion.

**TABLE 2:** Estimated prevalence for *Hemoplasma* spp. among Portuguese regions (n = 3959).

Region (Portugal)	Number of cats positive for *Hemoplasma* spp. qPCR	Estimated prevalence (%; 95%CI)
Alentejo	0	NA
Algavre	1	NA
Centro	52	6.2 (4.6‐7.9)
Lisboa	55	5.7 (4.2‐7.1)
Norte	103	4.7 (3.9‐5.8)

*Note*: Chi‐squared test *P* = .28.

Abbreviation: NA, not applicable.

Among the 4121 potential feline blood donors, 127 were positive for FeLV virus either by serology (antigen; n = 47), qPCR (DNA provirus; n = 62), or both diagnostic methods (n = 18) in 2022 leading to an overall estimated prevalence of 3.1% (95% CI: 2.6%‐3.6%). FIV serology was positive in 97 donors leading to an overall estimated prevalence of 2.3% (95% CI: 1.9%‐2.8%). Among these cats, 6 were positive for both FIV antibodies and FeLV detected with serology (n = 2), or qPCR (n = 3), or both diagnostic methods (n = 1).

### 3.3 *Hemoplasma* spp. association

The results of univariable and multivariable GEE models for the studied variables and hemoplasma positivity are shown in Table 3. After adjustment for age, breed, blood phenotype, Portuguese regions, and FIV positivity, *Hemoplasma* spp. qPCR positivity in blood donor units was significantly associated with male gender and FeLV positivity by serology and/or qPCR (Table 3). In the studied sample, *Hemoplasma* spp. qPCR was more frequently positive among blood units issued from male cats (137 (63.1%) units issued from a male cat in the *Hemoplasma* spp. qPCR positive units group vs 3466 (47.1%) in the negative group, OR = 1.9, 95% CI: 1.4‐2.6, *P* < 0.0001) and among blood units coinfected with FeLV (11 (5.1%) units coinfected with FeLV in the *Hemoplasma* spp. qPCR positive units group vs 129 (1.8%) in the negative group, OR = 2.8, 95% CI: 1.4‐5.6, *P* = .0023).

**TABLE 3:** Univariable and multivariable GEE regression analysis of clinical and epidemiological factors potentially associated with *Hemoplasma* spp. positivity in feline blood donation units (n = 7573).

Variables	Univariable analysis		Multivariable analysis	
	Crude OR (95% CI)	*P*	Adjusted OR (95% CI)	*P*
Sex (ref = female)	**1.9 (1.5‐2.6)**	**<.0001**	**1.9 (1.4‐2.6)**	**<.0001**
Age (years)	0.99 (0.94‐1.04)	.61	NA	NA
Breed (ref = nonpedigree)	0.52 (0.23‐1.2)	.13	NA	NA
Blood type (ref = A)	0.66 (0.27‐1.6)	.37	NA	NA
Portuguese region (ref = Centro)				
Lisboa	0.92 (0.62‐1.4)	.67	NA	NA
Norte	0.76 (0.54‐1.1)	.12	NA	NA
FeLV positivity (ref = negative)	**2.9 (1.5‐5.6)**	**.0018**	**2.8 (1.4‐5.6)**	**.0023**
FIV positivity (ref = negative)	2.1 (0.96‐4.8)	*.064*	2.0 (0.91‐4.6)	0.085

*Note*: Bolded values indicating 2‐tailed *P* < .05. Italicized value indicating 2‐tailed *P* < .1 were selected into the multiariable model.

Abbreviations: NA, not applicable (not included in multivariable model); Ref, reference for regression analysis.

The effect of seasonality was studied in a separate multivariable GEE model adjusted for gender and FeLV status. Blood units collected in Winter were more frequently positive for *Hemoplasma* spp. qPCR compared with the other seasons. *Hemoplasma* spp. qPCR was positive in 88 of 1838 (4.8%) units collected in winter (OR = 2.5, 95% CI: 1.7‐3.6, *P =* <.0001) in comparison with 38 of 1822 (2.1%) in Spring (OR = 1.1, 95% CI: 0.7‐1.6; *P* = .75), 53 of 1963 (2.7%) in Summer (OR = 1.4, 95% CI: 0.9‐2.0, *P* = .12) and 38 of 1950 (2.0%) in Autumn (=reference).

## 4 DISCUSSION

This study investigated the prevalence of hemoplasmas in potential feline blood donors in Portugal, Spain and Belgium in 2022. The overall estimated prevalence of hemoplasmas was 5.2% (95% CI: 4.5%‐5.9%). The prevalence estimated per country was 5.3% (95% CI: 4.6%‐5.9%) in Portugal and 2.9% (95% CI: 0.0%‐6.8%) in Spain. Prevalence estimation was not possible for Belgium. Variables associated with *Hemoplasma* spp. qPCR positivity in feline blood donor units were also investigated. Male gender, positivity for FeLV and collections performed during the Winter season were associated with a higher likelihood of *Hemoplasma* spp. positivity.

Prevalence of hemoplasmas in feline blood donors ranges from 3.7% in Portuguese and Spanish feline donors to 12.7% in feline donors in the United States.^21^, ^22^ The lower prevalence observed in Mediterranean European countries in the previous study could be attributed to the different study sample and different methodological laboratory methods used.^21^ In the present study, *Hemoplasma* spp. was detected using real‐time qPCR, while in previous study a multiplex conventional PCR was employed to detect the 3 most common feline hemoplasmas.^21^ Precautions should be taken when interpretating the low prevalence result obtained for Spanish potential feline donors in the present study; the small sample size from Spain (n = 70) compared with Portugal (n = 4034) prevented robust prevalence estimation for Spain with a large 95% confidence interval ranging from 0.0% to 6.8%. Prevalence estimation was not possible for Belgium because of the small sample size (n = 17).

Among cats that tested positive for *Hemoplasma* spp. via qPCR in 2022, 30 were tested for blood‐borne pathogens more than once over the year. Many of these cats were initially negative for hemoplasmas then tested positive later. A few cats tested positive twice. This finding highlights the interest of routine blood‐borne pathogens screening, particularly hemoplasmas, on every blood donation instead of annual screening as suggested in recent guidelines.^4^, ^5^, ^6^

Male gender, adult age, nonpedigree breed, outdoor access, blood genotype and coinfection with FIV and/or FeLV are associated with hemoplasmas positivity with conflicting results for some variables.^2^, ^8^, ^9^, ^10^, ^11^, ^12^, ^13^, ^14^, ^15^, ^16^, ^17^, ^18^, ^19^, ^20^ In the present study, a significant association was found for male gender and for FeLV positivity with *Hemoplasma* spp. qPCR positivity. Blood units issued from male cats or cats positive for FeLV by serology and/or PCR were more frequently positive for *Hemoplasma* spp. qPCR than blood units issued from female cats or cats negative for FeLV. The association between *Hemoplasma* spp. positivity and positivity for FIV was borderline (*P* < .1) in the univariate GEE model but did not reach significance in the multivariate model. This finding does not corroborate results from numerous previous studies,^8^, ^9^, ^11^, ^12^, ^13^, ^14^, ^15^, ^17^, ^18^ but agrees with 1 other.^2^ The conflicting results on the association between retroviral positivity and hemoplasmas observed in the literature might be explained by the variation in geographical prevalence of retroviruses. In areas where retroviruses are prevalent, there is a higher likelihood to identify an association with other infectious agents that can be transmitted via the same routes as retroviruses, such as social behaviors.^23^, ^24^ Indeed, interindividual aggressive interactions between cats have been suspected as routes of transmission for hemoplasmas.^25^, ^26^ The lower overall prevalence of FIV positivity (2.3%) compared with FeLV positivity (3.0%) in this study sample could explain the absence of association for FIV in the multivariate analysis. Another route of transmission that has been studied for hemoplasmas is vector‐borne transmission through arthropods, particularly fleas.^27^, ^28^ Vector‐borne transmission could explain the association between *Hemoplasma* spp. positivity and Winter season in this study. *Ctenocephalides felis* survival and development in the environment depends on minimal and maximal environmental temperatures favoring the egg to adult development ranging from 13°C to 32°C with extremes temperatures <3°C or >35°C having deleterious effects on survival.^29^, ^30^ Regarding environmental relative humidity (RH), complete development occurs from 50% to 92% RH, with increasing RH and decreasing temperatures favoring adult longevity.^29^ Consequently, a mild and humid climate, as observed in Portugal during the Winter months (https://www.ipma.pt/en/), might explain a higher proportion of fleas in the environment causing an increased possibility for vector‐borne transmission of hemoplasmas to occur in Winter. This is in contrast with previous studies that reported higher hemoplasma prevalence during summer months in Spain (Madrid) and in Northern Italy (Bologna).^9^, ^16^ Another explanation for the association between Winter season and *Hemoplasma* spp. qPCR positivity observed in the present study could be a modification of feline roaming habits, such as indoor confinement, favoring interindividual aggressive interactions and blood‐borne pathogens transmission. Finally, the removal of *Hemoplasma* positive cats from the donation program during the early months of the year corresponding to Winter could have had an impact on the results despite the use of a GEE model accounting for repeated blood donations on a same cat.

The present study had some limitations. First, because of its retrospective nature, missing information from the medical files were observed for some of the studied variables, particularly for the breed, which could have had an impact on statistical power. Additionally, some variables could have been interesting to include in the regression model, such as the lifestyle of the cat (indoor only vs restricted outdoor access; single‐housed vs multihoused), the external antiparasitic drug status, and the hematocrit. Such degree of precision in the data collection would have required a prospective longitudinal design, which was not intended here. Second, the *Hemoplasma* spp. qPCR used by the BSA—Animal Blood Bank did not allow to differentiate the subspecies of hemoplasmas, namely Mhf, CMhm and CMt, for specific prevalence estimation and statistical regression studies. Even if the true pathogenicity of CMhm and CMt has not been proven after blood transfusion, the BSA—Animal Blood Bank considers any hemoplasma as a potential infectious threat for the recipients. Therefore, subspecies identification is not routinely performed as blood units positive for *Hemoplasma* spp. qPCR are automatically discarded from the blood bank and the feline donor removed from the blood donation program. Moreover, our methodology did not assess the possibility for *Hemoplasma* spp. contamination in the reagents used for DNA extraction, which could potentially have resulted in false positive results. However, we maintained rigorous adherence to laboratory protocols during this critical phase, minimizing the likelihood of such contamination to happen. Lastly, the study sample size of Belgian potential feline donors was too small to perform prevalence estimation. A larger study with different inclusion criteria (eg, client‐owned cats admitted to hospital or stray cats) should be considered to gain insight into the prevalence of hemoplasma in that country.

In conclusion, among cats eligible for blood donation in Portugal, Spain and Belgium, 1 of 20 displayed a positive result for hemoplasmas in 2022 with an increased *Hemoplasma* spp. qPCR positivity observed in blood units positive for FeLV and issued from male cats. The seasonality for *Hemoplasma* spp. positivity, with an increased positivity observed in Winter in this study, remains to be elucidated. Finally, positive *Hemoplasma* spp. qPCR results identified in previously negative feline donors emphasizes the importance of testing on every donation unit instead of annually.

## CONFLICT OF INTEREST DECLARATION

Dr Ferrera Rui is a partner of the BSA and Dr Gommeren Kris is a partner of the Animal Blood Bank Benelux (part of the BSA). There are no other conflicts of interest to report.

## OFF‐LABEL ANTIMICROBIAL DECLARATION

Authors declare no off‐label use of antimicrobials.

## INSTITUTIONAL ANIMAL CARE AND USE COMMITTEE (IACUC) OR OTHER APPROVAL DECLARATION

Screening for *Hemoplasma* spp. and other infectious agents was performed on blood issued from blood donation as part as routine procedures performed by the Animal Blood Bank; no unnecessary procedures were done to blood donors. Every procedure at the blood bank required a signed informed owner consent.

## HUMAN ETHICS APPROVAL DECLARATION

Authors declare human ethics approval was not needed for this study.
